# Reasons for non-compliance to immunization among Fulani children aged between 0-11 months in the Vekovi community in Cameroon

**DOI:** 10.11604/pamj.2019.33.278.16900

**Published:** 2019-07-31

**Authors:** Tabe Armstrong Tambe, Xavier Tchetnya, Claude Ngwayu Nkfusai, Joyce Shirinde, Samuel Nambile Cumber

**Affiliations:** 1 Department of Public Health, School of Health Sciences, Catholic University of Central Africa, Box 1110, Yaounde, Cameroon; 2 Department of Microbiology and Parasitology, Faculty of Science, University of Buea, Buea, Cameroon; 3 Cameroon Baptist Convention Health Services (CBCHS), Yaounde, Cameroon; 4 School of Health Systems and Public Health, Faculty of Health Sciences, University of Pretoria Private Bag X323, Gezina, 0001, Pretoria, South Africa; 5 Faculty of Health Sciences, University of the Free State, Bloemfontein, South Africa; 6 Section for Epidemiology and Social Medicine, Department of Public Health, Institute of Medicine (EPSO), the Sahlgrenska Academy at University of Gothenburg, Box 414, SE –405 Gothenburg, Sweden

**Keywords:** Immunization, vaccination control, outbreak, diseases, prevention, community, non-compliance

## To the editors of the Pan African Medical Journal

**Figure 1:**
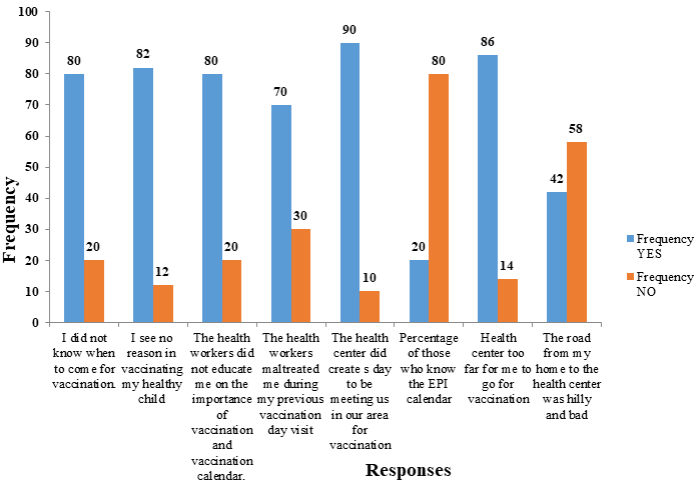
Distribution of respondents according to factors to non-compliance

With respect to vaccination, compliance rate is defined as the percentage of a distinct and eligible population with documented protection against specific vaccine-preventable diseases [1]. Immunization prevents illnesses, disability and deaths from vaccine-preventable diseases like cervical cancer and hepatitis B among others [1]. Global vaccination coverage stalled at 86% with no significant changes during the past years and the uptake of new underused vaccines is increasing with an additional 1.5 million deaths which could have been prevented with an increase in global vaccination. Unfortunately, an estimated 19.5 million infants still miss out basic vaccines [1]. To achieve effective immunization coverage, the Cameroon government deploys two main vaccination strategies which are the fixed techniques, where health workers stay in one spot, and the advanced technique where health workers moved from door-to-door, though stressful but increase vaccination coverage [2]. This cross-sectional study was carried out in the Vekovi Community Health Centre situated about 25kms from Shishong in Bui Division in the Northwest Region. A total of 100 participants constituting parents and guardians of children less than 11 months old attending the Infant Warefare Centre (IWC) were randomly recruited. Data was collected through a well-structured and pretested questionnaire designed to suit the objectives of the study. Data was registered and analyzed using Microsoft Excel version 10. Ethical clearance was obtained from the Institutional Research Ethics Committee for Human Health at the School of Health Sciences of the Catholic University of Central Africa while the administrative authorization was granted by the Delegate of Public Health for the Northwest Region. As revealed on Table 1, 34.0% of participants were between the age range of 21-30 years while 32.0% were between 31-40 years. The range above 40 years had the least representation (6.0%). Also, the study reveals that while 52% of the participants were married, 50% had farming as their main occupation. To say more, since 60% of the participants had no formal education and consequently could not read nor write, they didn't understand the importance of vaccination. This ties in with a study carried out in Ethiopia which revealed that due to illiteracy and ignorance, parents neglected vaccinating their children [3]. This study as illustrated in Figure 1, reveals that 80% of the participants did not know about the vaccination calendar nor did they about the importance of immunization (82 %). A huge percentage of the respondents (80%) were not motivated to vaccinate their healthy children given that the health personnel neither taught them the Expanded Program for Immunization (EPI) calendar during IWC nor the importance of vaccination. These findings corroborate studies carried out in rural communities in Nigeria and Arlington in 2009 in which health workers only provided general education rather than specific education on vaccination [4-5]. It was also observed that 70% of participants do not comply to IWC appointments because of past negative experiences with health care workers while distance was equally observed as a barrier to participants's compliance to immunization (86%). Moreover, poor topography prevented 42% of participants from respecting IWC appointments which can be justified by the fact that in most rural areas in Cameroon, roads to health facilities are very bad and worse worse during the rainy season. The door-to-door approach adopted by the government remains very challenging [6].

**Table 1:** The distribution of respondents according to response

Variable		Frequency	Percentage (%)
Age Range (Years)	11-20	28	28
	21-30	34	34
	31-40	32	32
	>40	6	6
	Total	100	100
Marital Status	Married	52	52
	Single	24	24
	Divorced	24	24
	Total	100	100
Level of Education	Primary	34	34
	Secondary	6	6
	High School	0	0
	None	60	60
	Total	100	100
Occupation	Farmer	50	50
	Student	6	6
	House Wife	39	39
	Others	5	5
	Total	100	100

## Conclusion

This paper concludes that most participants in the Vekovi Community in Cameroon, lack basic knowledge on the importance of immunization for their children, which is further compounded by parents and caregivers who have very poor knowledge on EPI calendar. Socio-cultural beliefs, poor service delivery, lack of formal education on the importance of immunization given by the health care workers, long distances to health facilities and bad roads, account for the huge gap in the immunization coverage for children within the ages 0-11 months Vekovi community. Therefore, there is need for government to put in place operative strategies to combat these barriers for effective immunization coverage. The researchers recommend that similar studies be carried out in other places. If findings are identical, it may influence policy makers to intervene positively to address the reasons for non-compliance to immunization in most communities.

## Competing interests

The authors declare no competing interests.
